# Changes in Thromboelastography to Predict Ecchymosis After Knee Arthroplasty: A Promising Guide for the Use of Anticoagulants

**DOI:** 10.3389/fsurg.2022.871776

**Published:** 2022-04-12

**Authors:** Yuelong Chen, Leilei Qin, Jianye Yang, Jiawei Wang, Jiaxing Huang, Xuan Gong, Ning Hu

**Affiliations:** ^1^Department of Respiratory and Critical Care Medicine, The First Affiliated Hospital of Chongqing Medical University, Chongqing, China; ^2^Department of Orthopaedics, The First Affiliated Hospital of Chongqing Medical University, Chongqing, China; ^3^Department of Orthopaedics, Fuling Central Hospital, Chongqing, China; ^4^Department of Outpatient Care, Chongqing General Hospital, Chongqing, China

**Keywords:** ecchymosis, total knee arthroplasty (TKA), thromboelastography (TEG), arthroplasty, anticoagulation

## Abstract

**Background:**

Ecchymosis is one of the worrisome complications after total knee arthroplasty (TKA) and interferes with functional rehabilitation. Current clinical guidelines do not provide individualized approaches for patients with ecchymoses.

**Methods:**

In this study, we used thromboelastography (TEG) to determine the coagulation state after TKA and to then explore markers that predict the occurrence of ecchymosis events after TKA. In our cohort, patients were divided into ecchymosis (*n* = 55) and non-ecchymosis (*n* = 137) groups according to whether ecchymosis events occurred after TKA. Rivaroxaban 10 mg/d was taken orally for thromboprophylaxis after surgery. All patients completed TEG testing. Correlation analysis was used to determine the risk factors for ecchymosis after TKA, and receiver operating characteristic (ROC) curves for variables with significant correlation were plotted.

**Results:**

In all, 55 of the 192 patients (28.65%) developed ecchymosis surrounding the surgical site. Multivariate analysis showed that hidden blood loss (OR = 1.003 and *p* = 0.005) and changes in the coagulation index (ΔCI) values (OR = 0.351 and *p* = 0.001) were risk factors for ecchymosis after TKA. Using the Youden index, 0.1805 was determined as the optimal threshold value of ΔCI for predicting the occurrence of ecchymosis, with a sensitivity of 74.55% and specificity of 72.99%. ΔCI is a promising marker as an alarm for the occurrence of ecchymosis after TKA.

**Trial Registration:**

The study was registered in the Chinese Clinical Trial Registry (ChiCTR1800017245). Registered name: The role of thrombelastography in monitoring the changes of coagulation function during perioperative period of arthroplasty. Registered 19 July 2018. http://www.chictr.org.cn/showproj.aspx?proj=29220

## Introduction

Total knee arthroplasty (TKA) is considered the most effective treatment for end-stage knee osteoarthritis (OA) (1). Due to the high prevalence of OA, TKA is a fairly common surgery. In the United States, the number of TKAs is projected to increase by 673% by 2030 (2). Venous thromboembolism (VTE) is a worrisome complication after TKA (3). Perioperative anticoagulant prophylaxis has been shown to reduce the incidence of postoperative VTE-related mortality and complications (4). Evidence-based guidelines recommend that patients undergoing TKA receive oral rivaroxaban for anticoagulant prophylaxis for 14 days (5, 6). However, postoperative bleeding complications associated with anticoagulation are not uncommon, especially with the widespread use of factor Xa inhibitors, in which the incidence of ecchymosis around the wound is as high as 13% (7).

The formation of postoperative ecchymosis around the wound is related to the use of anticoagulants (3). Ecchymosis around the surgical site can prolong the recovery time after TKA and may even lead to reoperation due to periprosthetic infection (8, 9). At present, there are still no clear guidelines for the balance between postoperative anticoagulation and bleeding (10). The use of anticoagulants should prevent VTE and avoid the occurrence of bleeding events. The monitoring of coagulation function has guiding value for the use of anticoagulants (11). Routine coagulation tests provide limited information about the quality of coagulation status (12, 13). Therefore, we need an alarm to predict the occurrence of ecchymosis events around the wound after TKA.

Thrombelastography (TEG) provides a comprehensive evaluation of blood viscoelastic properties and has potential value in predicting postoperative bleeding and thrombotic events (12, 14). Moreover, previous studies have shown that the change of coagulation index (ΔCI) value was a risk factor for patients with ecchymosis after TKA and was expected to guide personalized anticoagulant therapy (6). Thus, in this study, we sought to (1) explore whether the change in ΔCI can predict ecchymosis after TKA and (2) to calculate the threshold for predicting patients with ecchymosis based on ΔCI.

## Materials and Methods

From October 2018 to October 2020, we prospectively enrolled patients who were scheduled to undergo primary unilateral total knee arthroplasty (TKA) for knee OA. We excluded patients who (1) underwent bilateral TKA; (2) did not undergo TEG testing; (3) had a history of cardiovascular surgery, VTE or prior anticoagulant therapy; (4) were concomitant with coagulation disorders; (5) were treated with anticoagulation agents other than rivaroxaban; or (6) had incomplete medical records. According to the occurrence of ecchymosis after TKA, the patients were divided into ecchymosis and non-ecchymosis groups, ecchymosis was defined as subcutaneous extravasation of blood, without pain, swelling, or limited movement of the knee joint (6).

A tourniquet was used intraoperatively, and the tourniquet was loosened before the incision was closed. The anesthesiologist recorded the blood loss during the operation, mainly involving attracting blood from bottles and gauze. No drainage was used. All patients received standard physical therapy and rivaroxaban anticoagulant therapy for 14 days. Rivaroxaban (10 mg) was administered once daily starting 12 h after surgery, monitoring the occurrence of ecchymosis closely and discontinuing rivaroxaban once ecchymosis was observed. Venous blood was collected 1 day before surgery to obtain baseline hematocrit (HCT) and TEG values. The HCT and TEG values were monitored daily postoperatively until the patient was discharged. For discharged patients, investigators followed them up daily to see if there were any ecchymosis events. Once there were ecchymosis events, HCT and TEG tests were completed within 24 h, and anticoagulation therapy was stopped. TEG tests were performed by a TEG^®^ Hemostasis Analyzer (Hemonetics Corporation, Braintree, MA, USA).

All indicators of TEG (R-time, α-angle, maximum amplitude, and K-time) were recorded, and the CI was calculated using the formula CI = 0.1227(R) + 0.0092(K) + 0.1655(MA) – 0.0241(α) – 5.0220. We analyzed the ΔCI values (ΔCI = the postoperative CI value – the preoperative baseline CI value) for all patients. For patients without ecchymosis after TKA, we analyzed the maximum variation in CI relative to preoperative values; for patients with ecchymosis, we analyzed the ΔCI values between the day of ecchymosis occurrence and preoperatively. The gross (15) equation was used to calculate the volume of human erythrocytes and total blood loss. Then, hidden blood loss was the residual value of the total blood loss during the removal of intraoperative blood loss.

### Statistical Analysis

Statistical analysis was carried out with SPSS 24.0. Comparisons were made between the patients with and without ecchymosis. To clarify the values of factors related to ecchymosis in predicting the occurrence of ecchymosis events, the receiver operating characteristic (ROC) curve was established by MedCalc; the area under the ROC curve (AUC) was also calculated. The optimal cutoff values of each index for predicting the occurrence of ecchymosis events and the corresponding specificity and sensitivity were determined by Youden's J statistic. *P* < 0.05 was considered statistically significant.

## Results

A total of 192 patients who received a unilateral primary TKA were eligible for the study. In all, 55 of 192 patients (28.65%) developed ecchymosis surrounding the surgical site. There were no statistically significant differences between the two groups in terms of age (*p* = 0.125), sex (*p* = 0.480), or BMI (*P* = 0.085) (Table 1). During the follow-up, only three patients developed ecchymosis around the wound, which improved after anticoagulant treatment was stopped, and blood samples of these patients were obtained on the day that ecchymosis was observed.

**Table 1:** Demographic data for the study population.

**Variables**	**Non-ecchymosis group** **(*n* = 137)**	**Ecchymosis group** **(*n* = 55)**	***P*-value**
Age (years)	67.80 ± 10.787	65.16 ± 10.445	0.125
Gender (male/female)	40/97	13/42	0.480
Height	157.82 ± 7.026	157.48 ± 6.523	0.764
Weight	58.242 ± 9.6932	60.694 ± 10.1908	0.139
BMI (kg/m^2^)	23.355 ± 3.6025	24.4537 ± 4.0201	0.085

*BMI, body mass index. Variables are expressed as mean ± SD (standard deviation)*.

Total blood loss and hidden blood loss were significantly higher in the ecchymosis group compared to those in the non-ecchymosis group (Table 2). Compared to preoperative TKA, the average change in ΔCI in the ecchymosis group reached −0.5332 ± 1.1554, while the average change in ΔCI in the non-ecchymosis group reached 0.8490 ± 1.3344, thereby demonstrating a significant difference between the two groups (*p* = 0.001). The levels of total and hidden blood loss were 334.08 ± 125.71 ml and 319.36 ± 110.84 ml in the ecchymosis group, which were significantly higher than those in the non-ecchymosis group, with 287.8376 ± 109.4661 ml (*p* = 0.012) and 137.3048 ± 107.5984 ml (*p* < 0.001) for total blood loss and hidden blood loss, respectively. There were no significant differences in operative time (91.06 ± 10.406 vs. 94.69 ± 16.487 min) or intraoperative blood loss (31.53 ± 12.059 vs. 32.91 ± 12.045 ml) between the two groups (Table 2).

**Table 2:** Comparisons of variables of the ecchymosis group and non-ecchymosis group.

**Variables**	**Non-ecchymosis group**	**Ecchymosis group**	***P*-value**
ΔCI	0.8490 ± 1.3344	−0.5332 ± 1.1554	0.001
Operation time (min)	91.06 ± 10.406	94.69 ± 16.487	0.134
Total blood loss (mL)	287.8376 ± 109.4661	334.0836 ± 125.7136	0.012
Intraoperative blood loss (mL)	31.53 ± 12.059	32.91 ± 12.045	0.475
Hidden blood loss (mL)	137.3048 ± 107.5984	319.3564 ± 110.8418	<0.001

*ΔCI, change of coagulation index. Variables are expressed as mean ± SD (standard deviation)*.

We performed multivariate logistic regression analyses on potential risk factors for ecchymosis formation after TKA, including ΔCI, hidden blood loss, age, and BMI. The data suggested that ΔCI (OR: 0.351, 95% CI: 0.234, 0.526, and *p* = 0.001) and hidden blood loss (OR: 1.003, 95% CI: 1.000, 1.007, and *p* = 0.005) could be independent risk factors for the formation of ecchymosis, in addition to age, BMI, and total blood loss (Table 3).

**Table 3:** Risk factors for ecchymosis after TKA.

**Variables**	**OR (95% Confidence interval)**	***P-*value**
ΔCI	0.351 (0.234, 0.526)	0.001
Hidden blood loss (mL)	1.003 (1.000, 1.007)	0.005
Total blood loss (mL)		0.259
Age (years)		0.078
Gender (male/female)		0.479
BMI (kg/m^2^)		0.067

*ΔCI, change of coagulation index. BMI, body mass index. OR, odds ratio. TKA, total knee arthroplasty*.

**Figure 1:**
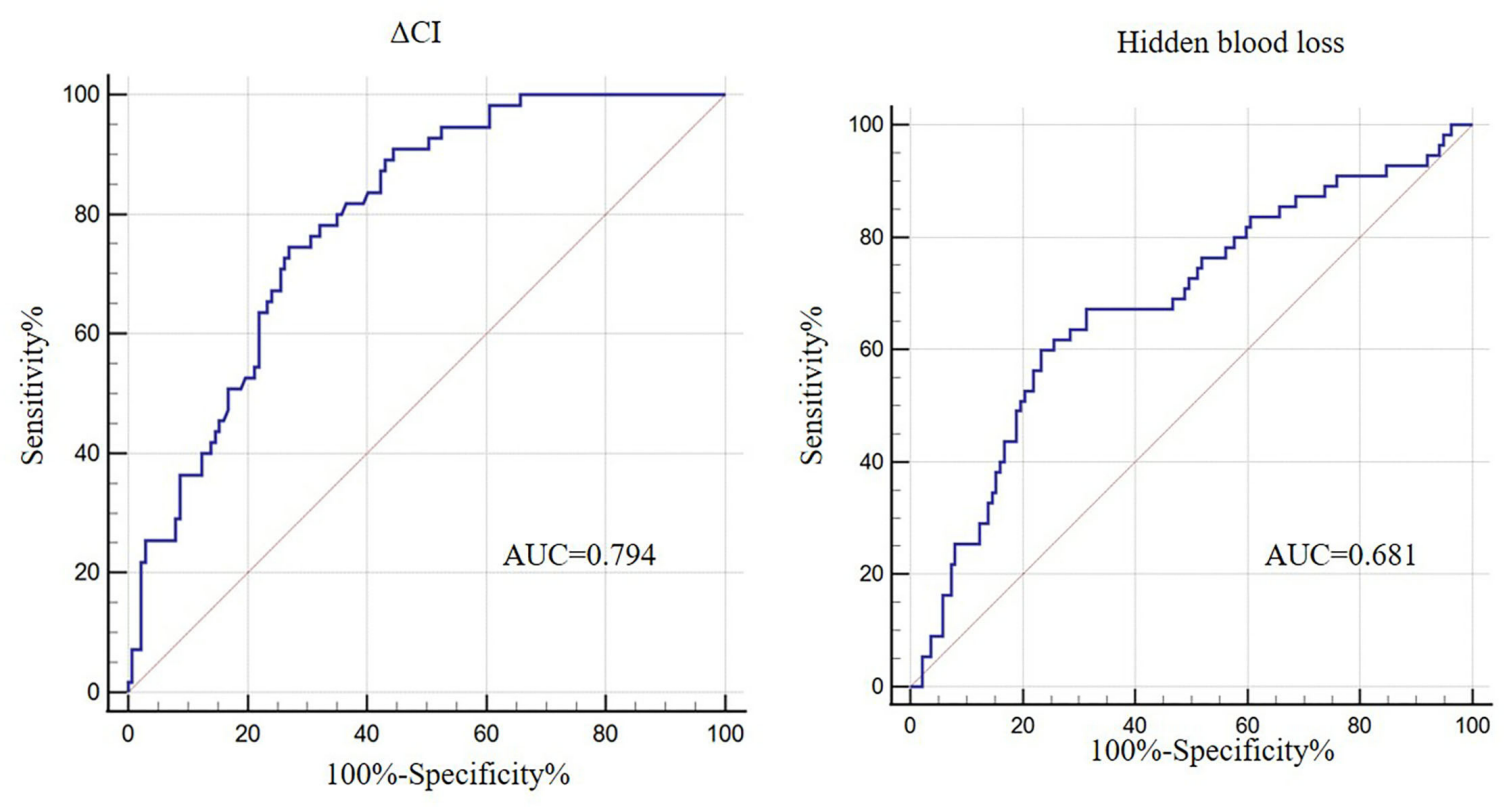
ROC curves of δCI and hidden blood loss in predicting the occurrence of ecchymosis.

To measure the value of CI and hidden blood loss in predicting the occurrence of ecchymosis, we plotted the ROC curves of the two variables (Figure 1). ΔCI discriminated between ecchymosis and non-ecchymosis with an AUC of 0.794 (95% CI: 0.730, 0.849). However, hidden blood loss did not exhibit a superior AUC of 0.681 (95% CI: 0.610, 0.746) (Figure 1).

As shown in Table 4, the threshold of CI was 0.1805, demonstrating a sensitivity of 74.55% (95% CI: 61.0, 85.3) and a specificity of 72.99% (95% CI: 64.7, 80.2) for predicting ecchymosis events after TKA. When the amount of hidden blood loss reached 311.63 ml the sensitivity and specificity for predicting the occurrence of ecchymosis were 60.00% (95% CI: 45.9, 73.0) and 76.64% (95% CI: 68.7, 83.4), respectively.

**Table 4:** Sensitivity, specificity, PPV, and NPV of variables for predicting ecchymosis after TKA.

**Parameters**	**AUC (95%CI)**	**Cut-off**	**Sensitivity (95%CI)**	**Specificity (95%CI)**	**PPV (%)**	**NPV (%)**	**LR+**	**LR–**	**Accuracy (%)**
ΔCI	0.794 (0.730, 0.849)	0.1805	74.55 (61.0, 85.3)	72.99 (64.7, 80.2)	52.6 (44.7, 60.3)	87.7 (81.8, 91.9)	2.76 (2.0, 3.8)	0.35 (0.2, 0.6)	73.4375
Hidden blood	0.681 (0.610, 0.746)	311.63	60.00 (45.9, 73.0)	76.64 (68.7, 83.4)	50.8 (41.5, 59.9)	82.7 (77.3, 87.0)	2.57 (1.8, 3.7)	0.52 (0.4, 0.7)	71.3542
loss (mL)									

*ΔCI, change of coagulation index. CI, confidence interval; PPV, positive predictive value; NPV, negative predictive value. TKA, total knee arthroplasty*.

## Discussion

To our knowledge, this study was the first attempt to use ΔCI to predict ecchymosis events after TKA and demonstrated its reliability. In our cohort, the ΔCI in the ecchymosis group was significantly higher than that in the non-ecchymosis group (*p* = 0.001) and was an independent risk factor (OR = 0.351, *p* = 0.001) for ecchymosis after TKA. The optimal cutoff value of ΔCI (0.1805) reflected maximal sensitivity (74.55%) and specificity (72.99%) to predict ecchymosis events after TKA. This study also found a significant correlation (OR = 1.003, *p* = 0.005) between hidden blood loss and ecchymosis events after TKA. Unfortunately, hidden blood loss showed unsatisfactory sensitivity in predicting the occurrence of ecchymosis events. Hidden blood loss is generally defined as blood deposited in the joint space and blood seeping into the tissue (16, 17). However, when the volume of hidden blood loss was not sufficient, the blood in the tissue may have been absorbed by the body before it penetrated the mucosa of the skin, preventing the formation of ecchymosis.

Damage to the vascular wall at the surgical site provides a possibility for the formation of ecchymosis, but it may not be the only cause (18). The effect of anticoagulants on the formation of ecchymosis cannot be ignored. In the absence of thromboprophylaxis treatment, the incidence of VTE after TKA can be as high as 40–84% (19). However, anticoagulant chemoprophylaxis puts patients at risk for bleeding after TKA. Bleeding complications (including ecchymosis) following total joint arthroplasty are not acceptable, as they can lead to more important complications, such as infection, wound healing problems, dysfunction and loosening of the joints, with a high likelihood of affecting the surgical outcome (20). Rivaroxaban, a direct oral factor Xa inhibitor, is commonly prescribed for the prevention of VTE after TKA due to its effectiveness, high safety and convenience of use (3, 21). However, evidence has shown that rivaroxaban use also increases the risk of postoperative bleeding (7). This poses a challenge to the balance between anticoagulation and bleeding prevention. Therefore, accurate monitoring of coagulation status and early prediction of ecchymosis events are the key links of individual anticoagulation.

TEG is commonly used to evaluate the viscoelastic properties of a patient's whole blood during surgery (22). As early as 2009, Kashuk et al. (14) showed the role of TEG in identifying hypercoagulability and predicting thromboembolic events in surgical patients. Previous studies have attempted to apply ΔR to adjust the use of anticoagulants, but the data showed that ΔR does not have this capability (11). The possible explanation is that the clotting process involved in the formation of a thrombus or fibrinolysis is complex, and it is unreliable to represent the whole process by a single point of the clotting process. Therefore, in this study, we analyzed and compared the preoperative and postoperative changes in the comprehensive evaluation index CI. Through the analysis of two cohorts with or without ecchymosis after TKA, we found a high correlation between ΔCI and ecchymosis, which was consistent with previous research results (6). In addition, we also confirmed that, when the CI was lower than 0.1805, it was a warning that the body was in a hypocoagulable state, and the probability of ecchymosis events was as high as 73.44%.

The patients were prospectively recruited, with each patient followed for at least 2 weeks. The 2-week follow-up covered the entire course of the patient's anticoagulant use, and it was assured that the patient's hemodynamics had stabilized by the end of the follow-up (9).

Some limitations need to be noted in this study. First, this study was performed in a single center, and more regional studies are needed to support our conclusions. We are, in a follow-up study, examining this issue. Second, the patients who underwent TKA were elderly and had different types of underlying diseases. Age and underlying diseases may also be risk factors for the occurrence of ecchymosis (23, 24). In this study, there was no subgroup analyses on the types of diseases and ages of patients, so the results of the study may be biased. Last, most of the patients were hospitalized for 5 days after surgery. Except for the patients with ecchymosis, blood samples were collected on the day that ecchymosis appeared, and the patients without ecchymosis only had TEG testing during hospitalization. Therefore, it was difficult to ensure that the maximum CI we monitored was the maximum in the non-ecchymosis group.

This study, to our knowledge, is the first to demonstrate that ΔCI in TEG parameters can be used as a predictor of ecchymosis events after TKA, and the optimal cutoff value was 0.1805. On the basis of our findings, we believe that ΔCI is a promising indicator to guide the use of anticoagulants early after TKA. When the ΔCI was lower than 0.1805, it was reasonable to consider stopping the use of rivaroxaban to avoid the occurrence of ecchymosis events. Of course, more research is needed to verify and confirm the reliability of this prediction.

## Data Availability Statement

The raw data supporting the conclusions of this article will be made available by the authors, without undue reservation.

## Ethics Statement

The studies involving human participants were reviewed and approved by Institutional Review Board and Hospital Ethics Committee of Chongqing Medical University. Written informed consent to participate in this study was provided by the participants' legal guardian/next of kin.

## Author Contributions

NH and XG contributed to the experimental ideas, design of this study, examined, and revised the contents of the manuscript. YC drafted the manuscript. LQ, JY, and JH collected and analyzed the data. JW performed the statistical analysis. All authors approved the final submitted version.

## Funding

This research was supported by the National Natural Science Foundation of China (82072443) and the Chongqing Technology Innovation and Application Development Special General Project (no: cstc2020jscx-msxmX0094).

## Conflict of Interest

The authors declare that the research was conducted in the absence of any commercial or financial relationships that could be construed as a potential conflict of interest.

## Publisher's Note

All claims expressed in this article are solely those of the authors and do not necessarily represent those of their affiliated organizations, or those of the publisher, the editors and the reviewers. Any product that may be evaluated in this article, or claim that may be made by its manufacturer, is not guaranteed or endorsed by the publisher.
